# The American Medical Association

**Published:** 1888-06

**Authors:** P. R. Cortelyou


### THE AMERICAN MEDICAL ASSOCIATION.

REPORTED BY P. R. CORTELYOU, M. D.

The Thirty-ninth Annual Meeting of the American Medical Association was held in Cincinnati, Ohio, commencing May 8th, and continuing in session until May 11th. It proved to be a very successful and harmonious meeting, and was largely attended, many leading men in the profession, from all parts of the country, being present. Among some of the notable ones may be mentioned Drs. N. S. Davis, of Chicago; Bartholow, of Philadelphia; Wm. Pepper, of Philadelphia; Shoemaker, Dulles, Battey, Ingalls, Goodell, and many others.

The opening address of the distinguished President, A. Y. P. Garnett, of Washington, D. C., was full of thought; his views in regard to higher medical education, and increased efforts to raise the standard of graduation in medicine, were enthusiastically received.

The address on "General Medicine," by Dr. Roberts Bartholow, of Philadelphia, was brilliant in style, and complete in all respects,

Dr. E. A. Wood, of Pittsburgh, Pennsylvania, read Report on Dietetics, and it was received with much applause. He thought that in the vast number of prepared foods and digestive preparations, we were in danger of leaving little for nature to do, and so defeating the end in view, weakening instead of strengthening the digestive organs.

The address of the venerable Dr. E. M. Moore, of Rochester, New York, on "General Surgery," was excellent in all particulars, and minutely described all the advantages to physician and patient, which resulted from the adoption of the antiseptic methods in all surgical operations.

The work done in the various sections was of a high order, and showed the profession to be alive to the greater advancement of the healing art in all its branches.

In the Section of Practical Medicine, pneumonia was fully discussed, and the treatment of the present day compared with that of twenty-five years ago; and some were inclined to believe that the blood letting of those days showed as good, if not better results, than the present method of antipyretics and stimulants.

Dr. Wm. Pepper, of Philadelphia, spoke on the Diseases of Stomach, more particularly of carcinoma, stating that in these cases there was an absence of hydrochloric acid in the digestive act, and that this fact could be very readily determined by chemical means of diagnosis in those doubtful cases.

The discussion of this subject, by Dr. Whitaker, of Cincinnati, and Dr. Shattuck, of Boston, was very instructive.

A paper on cerebro-spinal meningitis was read by Dr. J. McF. Gaston.

The Section on Surgery and Anatomy, devoted much of its time to abdominal surgery, and the diseases of the Apendix Vermiformis were fully investigated, and the best course of procedure in those cases. A paper on this subject was read by Dr. J. McF. Gaston.

The experiments of Dr. N. Senn, of Milwaukee, Wisconsin, in ascertaining the existence of visceral injury of the gastro intestinal canal, in penetrating wounds of the abdomen, by insufflation of hydrogen gas, were novel, and convincing. He shot a dog in the abdomen, then inflated intestines with hydrogen gas, and with a lighted match held to the wound showed the escape of the gas by its being ignited, thus proving that the intestines had been punctured.

The Section on Diseases of Children, also had much that was interesting and instructive under discussion. The subject of Infant Feeding and Foods was exhaustively treated by Drs. E. W. Earle, of Chicago; Whittaker, Love, Ripley, Lanabee, and others. The gist of the whole matter was, that nothing could take the place of mother's milk; and that the next best was good cow's milk, partly peptonized, and the caseine thus made light and flocculent.

Diphtheria and Membranous Croup a so received their full consideration from the Section. Intubation of the larynx in these cases received strong endorsement, and was considered in many cases preferable to tracheotomy and as giving as favorable results.

Many more important subjects were handled in the various sections, but time and space would fail to mention them all.

The meetings were held in Music Hall, and ample room was furnished for all the Sections to meet in the same building, thus making it very convenient for one to visit the different Sections. The social features were very pleasant. A reception was held on Tuesday night at the Burnett House, Wednesday night at the Eden Art Museum, Thursday night a musical concert was given at Music Hall, by the Apollo Club, of Cincinnati, at which four thousand were present, and enjoyed the rich musical feast.

Dr. W. W. Dawson, of Cincinnati, was elected as president for the coming year; Dr. J. W. Bailey, of Gainesville, Georgia, one of the Vice-Presidents, and Dr. J. McFadden Gaston, of Atlanta, one of the Judicial Committee. Newport, R. I., was selected for the next place of meeting in June, 1889.

In reviewing the session as a whole, we are satisfied that it was one of the most successful that the Association has ever held, and that the American Medical Association is a grand factor in advancing medical science in this country.

